# [*N*′-(3-Eth­oxy-2-oxidobenzyl­idene-κ*O* ^2^)-4-methyl­benzohydrazidato-κ^2^ *O*,*N*′](methano­lato-κ*O*)oxidovanadium(V)

**DOI:** 10.1107/S1600536811041547

**Published:** 2011-10-12

**Authors:** Chen-Yi Wang, Xiang Wu, Feng Cao, Cai-Jun Yuan

**Affiliations:** a Department of Chemistry, Huzhou University, Huzhou 313000, People’s Republic of China

## Abstract

The title oxidovanadium(V) complex, [V(C_17_H_16_N_2_O_3_)(CH_3_O)O], was obtained by the reaction of 3-eth­oxy-2-hy­droxy­benzaldehyde, 4-methyl­benzohydrazide and vanadyl sulfate in methanol. The V^V^ atom is coordinated by the *O*,*N*,*O*′-tridentate Schiff base ligand, one methano­late O atom and one oxide O atom, forming a distorted VO_4_N square-pyramidal coordination geometry. The oxide O atom lies at the apex of the square pyramid and the N atom of the ligand and the methano­late O atom are *trans*. The dihedral angle between the benzene rings of the ligand is 1.8 (3)°.

## Related literature

For background to Schiff base complexes, see: Wang (2009 ▶); Wang & Ye (2011 ▶). For similar vanadium(V) complexes, see: Wang *et al.* (2011 ▶); Deng *et al.* (2005 ▶); Gao *et al.* (2005 ▶); Huo *et al.* (2004 ▶).
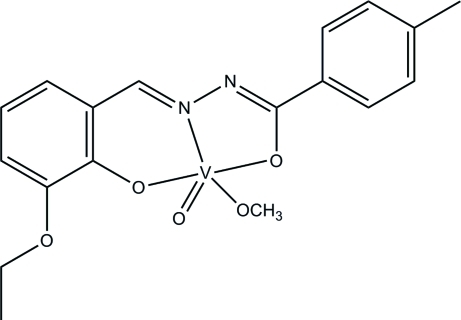


## Experimental

### 

#### Crystal data

[V(C_17_H_16_N_2_O_3_)(CH_3_O)O]
*M* *_r_* = 394.29
Monoclinic, 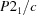

*a* = 7.6954 (16) Å
*b* = 28.345 (3) Å
*c* = 8.3877 (18) Å
β = 105.175 (2)°
*V* = 1765.8 (6) Å^3^
*Z* = 4
Mo *K*α radiation
μ = 0.59 mm^−1^
*T* = 298 K
0.30 × 0.27 × 0.27 mm

#### Data collection

Bruker SMART CCD diffractometer
Absorption correction: multi-scan (*SADABS*; Sheldrick, 1996 ▶) *T*_min_ = 0.842, *T*_max_ = 0.856
14022 measured reflections
4044 independent reflections
2986 reflections with *I* > 2σ(*I*)
*R*_int_ = 0.039

#### Refinement

*R*[*F* ^2^ > 2σ(*F* ^2^)] = 0.045
*wR*(*F* ^2^) = 0.110
*S* = 1.05
4044 reflections
238 parameters
H-atom parameters constrained
Δρ_max_ = 0.27 e Å^−3^
Δρ_min_ = −0.30 e Å^−3^

### 

Data collection: *SMART* (Bruker, 1998 ▶); cell refinement: *SAINT* (Bruker, 1998 ▶); data reduction: *SAINT*; program(s) used to solve structure: *SHELXS97* (Sheldrick, 2008 ▶); program(s) used to refine structure: *SHELXL97* (Sheldrick, 2008 ▶); molecular graphics: *SHELXTL* (Sheldrick, 2008 ▶); software used to prepare material for publication: *SHELXTL*.

## Supplementary Material

Crystal structure: contains datablock(s) global, I. DOI: 10.1107/S1600536811041547/hb6443sup1.cif

Structure factors: contains datablock(s) I. DOI: 10.1107/S1600536811041547/hb6443Isup2.hkl

Additional supplementary materials:  crystallographic information; 3D view; checkCIF report

## Figures and Tables

**Table 1:** Selected bond lengths (Å)

V1—O5	1.5813 (17)
V1—O4	1.7499 (17)
V1—O1	1.8326 (16)
V1—O3	1.9170 (16)
V1—N1	2.1031 (19)

**Fig. 1.**
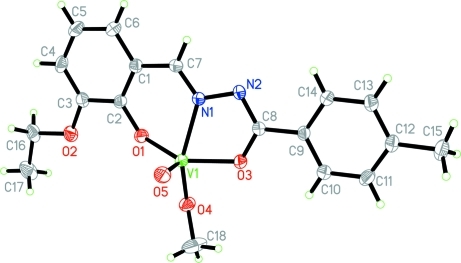
The molecular structure of the title compound, showing displacement ellipsoids drawn at the 30% probability level.

## supplementary crystallographic information

### Comment

As part of our investigations into new Schiff base complexes and urease inhibition (Wang & Ye, 2011; Wang, 2009), we have synthesized the title compound, (I), a new mononuclear oxovanadium(V) complex, Fig. 1. The V atom in the complex is five-coordinated by the O,N,O-tridentate Schiff base ligand, one methanolate O atom, and one oxide O atom, forming a distorted square-pyramidal geometry. The oxide O atom lies on the apical position of the square-pyramidal geometry. The dihedral angle between the two benzene rings is 1.8 (3)°. The V–O and V–N bond lengths (Table 1) are typical and are comparable with those observed in other similar vanadium complexes (Wang *et al.*, 2011; Deng *et al.*, 2005; Gao *et al.*, 2005; Huo *et al.*, 2004).

### Experimental

3-Ethoxy-2-hydroxybenzaldehyde (1.0 mmol, 0.17 g), 4-methylbenzohydrazide (1.0 mmol, 0.15 g), and vanadyl sulfate (1.0 mmol, 0.16 g) were dissolved in methanol (30 ml). The mixture was stirred at room temperature for 10 min to give a clear brown solution. After keeping the solution in air for a week, brown block-shaped crystals were formed at the bottom of the vessel.

### Refinement

Hydrogen atoms were placed in geometrically idealized positions and constrained to ride on their parent atoms, with C—H distances in the range 0.93–0.97 Å, and with *U*_iso_(H) set at 1.2 or 1.5*U*_eq_(C).

### Crystal data

**Table:**

[V(C_17_H_16_N_2_O_3_)(CH_3_O)O]	*F*(000) = 816
*M**_r_* = 394.29	*D*_x_ = 1.483 Mg m^−^^3^
Monoclinic, *P*2_1_/*c*	Mo *K*α radiation, λ = 0.71073 Å
Hall symbol: -P 2ybc	Cell parameters from 3023 reflections
*a* = 7.6954 (16) Å	θ = 2.6–25.0°
*b* = 28.345 (3) Å	µ = 0.59 mm^−^^1^
*c* = 8.3877 (18) Å	*T* = 298 K
β = 105.175 (2)°	Block, brown
*V* = 1765.8 (6) Å^3^	0.30 × 0.27 × 0.27 mm
*Z* = 4	

### Data collection

**Table:**

Bruker SMART CCD diffractometer	4044 independent reflections
Radiation source: fine-focus sealed tube	2986 reflections with *I* > 2σ(*I*)
graphite	*R*_int_ = 0.039
ω scans	θ_max_ = 27.5°, θ_min_ = 2.6°
Absorption correction: multi-scan (*SADABS*; Sheldrick, 1996)	*h* = −9→9
*T*_min_ = 0.842, *T*_max_ = 0.856	*k* = −36→35
14022 measured reflections	*l* = −10→10

### Refinement

**Table:**

Refinement on *F*^2^	Primary atom site location: structure-invariant direct methods
Least-squares matrix: full	Secondary atom site location: difference Fourier map
*R*[*F*^2^ > 2σ(*F*^2^)] = 0.045	Hydrogen site location: inferred from neighbouring sites
*wR*(*F*^2^) = 0.110	H-atom parameters constrained
*S* = 1.05	*w* = 1/[σ^2^(*F*_o_^2^) + (0.0473*P*)^2^ + 0.4204*P*] where *P* = (*F*_o_^2^ + 2*F*_c_^2^)/3
4044 reflections	(Δ/σ)_max_ = 0.001
238 parameters	Δρ_max_ = 0.27 e Å^−^^3^
0 restraints	Δρ_min_ = −0.30 e Å^−^^3^

### Special details

**Table:**

Geometry. All e.s.d.'s (except the e.s.d. in the dihedral angle between two l.s. planes) are estimated using the full covariance matrix. The cell e.s.d.'s are taken into account individually in the estimation of e.s.d.'s in distances, angles and torsion angles; correlations between e.s.d.'s in cell parameters are only used when they are defined by crystal symmetry. An approximate (isotropic) treatment of cell e.s.d.'s is used for estimating e.s.d.'s involving l.s. planes.
Refinement. Refinement of *F*^2^ against ALL reflections. The weighted *R*-factor *wR* and goodness of fit *S* are based on *F*^2^, conventional *R*-factors *R* are based on *F*, with *F* set to zero for negative *F*^2^. The threshold expression of *F*^2^ > σ(*F*^2^) is used only for calculating *R*-factors(gt) *etc*. and is not relevant to the choice of reflections for refinement. *R*-factors based on *F*^2^ are statistically about twice as large as those based on *F*, and *R*- factors based on ALL data will be even larger.

### Fractional atomic coordinates and isotropic or equivalent isotropic displacement parameters (Å^2^)

**Table:**

	*x*	*y*	*z*	*U*_iso_*/*U*_eq_	
V1	0.23672 (5)	0.414915 (13)	0.47578 (5)	0.03413 (14)	
N1	0.1850 (2)	0.34208 (6)	0.4771 (2)	0.0333 (4)	
N2	0.0915 (3)	0.32243 (7)	0.3264 (2)	0.0386 (5)	
O1	0.3948 (2)	0.40199 (5)	0.67570 (19)	0.0407 (4)	
O2	0.5797 (2)	0.42530 (6)	0.9805 (2)	0.0477 (5)	
O3	0.1583 (2)	0.39594 (6)	0.24884 (19)	0.0411 (4)	
O4	0.3495 (2)	0.46251 (6)	0.4152 (2)	0.0478 (4)	
O5	0.0567 (2)	0.43456 (6)	0.5082 (2)	0.0479 (4)	
C1	0.3177 (3)	0.32665 (8)	0.7653 (3)	0.0342 (5)	
C2	0.4022 (3)	0.37085 (8)	0.7972 (3)	0.0340 (5)	
C3	0.5005 (3)	0.38212 (9)	0.9597 (3)	0.0386 (6)	
C4	0.5096 (3)	0.34910 (10)	1.0833 (3)	0.0466 (6)	
H4	0.5724	0.3564	1.1910	0.056*	
C5	0.4279 (3)	0.30551 (10)	1.0504 (3)	0.0498 (7)	
H5	0.4376	0.2839	1.1358	0.060*	
C6	0.3330 (3)	0.29378 (9)	0.8939 (3)	0.0422 (6)	
H6	0.2789	0.2643	0.8726	0.051*	
C7	0.2177 (3)	0.31382 (8)	0.6028 (3)	0.0350 (5)	
H7	0.1731	0.2832	0.5860	0.042*	
C8	0.0825 (3)	0.35451 (8)	0.2130 (3)	0.0336 (5)	
C9	−0.0137 (3)	0.34495 (8)	0.0398 (3)	0.0324 (5)	
C10	−0.0244 (3)	0.37956 (9)	−0.0791 (3)	0.0411 (6)	
H10	0.0317	0.4085	−0.0499	0.049*	
C11	−0.1182 (3)	0.37113 (9)	−0.2405 (3)	0.0449 (6)	
H11	−0.1248	0.3947	−0.3188	0.054*	
C12	−0.2024 (3)	0.32857 (9)	−0.2889 (3)	0.0388 (6)	
C13	−0.1894 (3)	0.29402 (9)	−0.1695 (3)	0.0439 (6)	
H13	−0.2440	0.2649	−0.1994	0.053*	
C14	−0.0972 (3)	0.30194 (8)	−0.0072 (3)	0.0393 (6)	
H14	−0.0911	0.2783	0.0710	0.047*	
C15	−0.3043 (4)	0.32044 (11)	−0.4656 (3)	0.0559 (7)	
H15A	−0.3865	0.3461	−0.5028	0.084*	
H15B	−0.3704	0.2914	−0.4740	0.084*	
H15C	−0.2212	0.3186	−0.5331	0.084*	
C16	0.6757 (4)	0.43846 (10)	1.1461 (3)	0.0546 (7)	
H16A	0.7681	0.4153	1.1917	0.065*	
H16B	0.5935	0.4399	1.2161	0.065*	
C17	0.7590 (4)	0.48552 (11)	1.1390 (4)	0.0727 (10)	
H17A	0.8465	0.4833	1.0761	0.109*	
H17B	0.8169	0.4959	1.2490	0.109*	
H17C	0.6674	0.5078	1.0873	0.109*	
C18	0.2882 (4)	0.50506 (11)	0.3339 (4)	0.0795 (11)	
H18A	0.1982	0.4985	0.2333	0.119*	
H18B	0.3873	0.5213	0.3090	0.119*	
H18C	0.2373	0.5244	0.4040	0.119*	

### Atomic displacement parameters (Å^2^)

**Table:**

	*U*^11^	*U*^22^	*U*^33^	*U*^12^	*U*^13^	*U*^23^
V1	0.0416 (2)	0.0292 (2)	0.0292 (2)	−0.00241 (17)	0.00511 (16)	0.00033 (17)
N1	0.0382 (10)	0.0303 (10)	0.0294 (10)	−0.0005 (8)	0.0050 (8)	−0.0002 (8)
N2	0.0492 (12)	0.0339 (11)	0.0280 (10)	−0.0048 (9)	0.0018 (8)	−0.0042 (8)
O1	0.0486 (10)	0.0349 (9)	0.0324 (9)	−0.0063 (7)	−0.0004 (7)	0.0007 (7)
O2	0.0515 (10)	0.0465 (11)	0.0352 (10)	0.0005 (8)	−0.0063 (8)	−0.0065 (8)
O3	0.0569 (10)	0.0346 (9)	0.0302 (9)	−0.0100 (8)	0.0086 (7)	−0.0009 (7)
O4	0.0544 (10)	0.0414 (10)	0.0427 (10)	−0.0136 (8)	0.0042 (8)	0.0062 (8)
O5	0.0524 (10)	0.0444 (10)	0.0480 (11)	0.0074 (8)	0.0151 (8)	0.0006 (8)
C1	0.0334 (11)	0.0370 (13)	0.0315 (12)	0.0051 (10)	0.0071 (9)	0.0009 (10)
C2	0.0355 (12)	0.0344 (12)	0.0304 (12)	0.0080 (10)	0.0057 (9)	0.0025 (10)
C3	0.0381 (12)	0.0412 (14)	0.0339 (13)	0.0079 (10)	0.0049 (10)	−0.0038 (10)
C4	0.0488 (14)	0.0608 (18)	0.0259 (13)	0.0095 (13)	0.0021 (10)	0.0025 (12)
C5	0.0573 (16)	0.0549 (17)	0.0357 (14)	0.0058 (13)	0.0093 (12)	0.0144 (13)
C6	0.0450 (13)	0.0436 (15)	0.0381 (14)	0.0015 (11)	0.0110 (11)	0.0085 (11)
C7	0.0397 (12)	0.0291 (12)	0.0353 (13)	−0.0004 (9)	0.0084 (10)	0.0010 (10)
C8	0.0378 (12)	0.0325 (12)	0.0309 (12)	−0.0002 (10)	0.0094 (9)	−0.0009 (10)
C9	0.0358 (12)	0.0339 (12)	0.0277 (12)	0.0035 (9)	0.0086 (9)	−0.0019 (10)
C10	0.0516 (14)	0.0368 (14)	0.0353 (13)	−0.0018 (11)	0.0124 (11)	−0.0004 (11)
C11	0.0546 (15)	0.0486 (15)	0.0337 (14)	0.0082 (12)	0.0157 (11)	0.0093 (12)
C12	0.0367 (12)	0.0491 (15)	0.0304 (13)	0.0055 (11)	0.0083 (10)	−0.0018 (11)
C13	0.0478 (14)	0.0448 (15)	0.0359 (14)	−0.0070 (11)	0.0054 (11)	−0.0056 (11)
C14	0.0466 (13)	0.0382 (14)	0.0306 (13)	−0.0010 (11)	0.0058 (10)	0.0024 (10)
C15	0.0592 (16)	0.071 (2)	0.0323 (14)	0.0095 (15)	0.0031 (12)	−0.0006 (14)
C16	0.0515 (15)	0.0630 (18)	0.0385 (15)	0.0086 (14)	−0.0072 (12)	−0.0153 (13)
C17	0.071 (2)	0.062 (2)	0.067 (2)	−0.0001 (16)	−0.0148 (16)	−0.0230 (17)
C18	0.069 (2)	0.062 (2)	0.092 (3)	−0.0136 (16)	−0.0071 (18)	0.0393 (18)

### Geometric parameters (Å, °)

**Table:**

V1—O5	1.5813 (17)	C8—C9	1.473 (3)
V1—O4	1.7499 (17)	C9—C10	1.386 (3)
V1—O1	1.8326 (16)	C9—C14	1.386 (3)
V1—O3	1.9170 (16)	C10—C11	1.378 (3)
V1—N1	2.1031 (19)	C10—H10	0.9300
N1—C7	1.295 (3)	C11—C12	1.380 (3)
N1—N2	1.396 (2)	C11—H11	0.9300
N2—C8	1.305 (3)	C12—C13	1.385 (3)
O1—C2	1.338 (3)	C12—C15	1.502 (3)
O2—C3	1.358 (3)	C13—C14	1.379 (3)
O2—C16	1.441 (3)	C13—H13	0.9300
O3—C8	1.311 (3)	C14—H14	0.9300
O4—C18	1.405 (3)	C15—H15A	0.9600
C1—C2	1.405 (3)	C15—H15B	0.9600
C1—C6	1.407 (3)	C15—H15C	0.9600
C1—C7	1.426 (3)	C16—C17	1.488 (4)
C2—C3	1.412 (3)	C16—H16A	0.9700
C3—C4	1.384 (3)	C16—H16B	0.9700
C4—C5	1.381 (4)	C17—H17A	0.9600
C4—H4	0.9300	C17—H17B	0.9600
C5—C6	1.366 (3)	C17—H17C	0.9600
C5—H5	0.9300	C18—H18A	0.9600
C6—H6	0.9300	C18—H18B	0.9600
C7—H7	0.9300	C18—H18C	0.9600
			
O5—V1—O4	107.58 (9)	C10—C9—C14	118.8 (2)
O5—V1—O1	108.34 (9)	C10—C9—C8	120.0 (2)
O4—V1—O1	99.14 (8)	C14—C9—C8	121.2 (2)
O5—V1—O3	101.98 (8)	C11—C10—C9	120.1 (2)
O4—V1—O3	88.79 (7)	C11—C10—H10	119.9
O1—V1—O3	144.43 (8)	C9—C10—H10	119.9
O5—V1—N1	99.74 (8)	C10—C11—C12	121.7 (2)
O4—V1—N1	150.14 (8)	C10—C11—H11	119.1
O1—V1—N1	83.11 (7)	C12—C11—H11	119.1
O3—V1—N1	73.70 (7)	C11—C12—C13	117.8 (2)
C7—N1—N2	115.75 (19)	C11—C12—C15	120.6 (2)
C7—N1—V1	127.94 (16)	C13—C12—C15	121.7 (2)
N2—N1—V1	116.11 (13)	C14—C13—C12	121.3 (2)
C8—N2—N1	107.35 (18)	C14—C13—H13	119.3
C2—O1—V1	135.60 (15)	C12—C13—H13	119.3
C3—O2—C16	117.1 (2)	C13—C14—C9	120.3 (2)
C8—O3—V1	118.96 (14)	C13—C14—H14	119.8
C18—O4—V1	132.35 (17)	C9—C14—H14	119.8
C2—C1—C6	120.2 (2)	C12—C15—H15A	109.5
C2—C1—C7	121.1 (2)	C12—C15—H15B	109.5
C6—C1—C7	118.7 (2)	H15A—C15—H15B	109.5
O1—C2—C1	121.2 (2)	C12—C15—H15C	109.5
O1—C2—C3	119.4 (2)	H15A—C15—H15C	109.5
C1—C2—C3	119.4 (2)	H15B—C15—H15C	109.5
O2—C3—C4	125.5 (2)	O2—C16—C17	108.1 (2)
O2—C3—C2	115.9 (2)	O2—C16—H16A	110.1
C4—C3—C2	118.6 (2)	C17—C16—H16A	110.1
C5—C4—C3	121.6 (2)	O2—C16—H16B	110.1
C5—C4—H4	119.2	C17—C16—H16B	110.1
C3—C4—H4	119.2	H16A—C16—H16B	108.4
C6—C5—C4	120.7 (2)	C16—C17—H17A	109.5
C6—C5—H5	119.6	C16—C17—H17B	109.5
C4—C5—H5	119.6	H17A—C17—H17B	109.5
C5—C6—C1	119.5 (2)	C16—C17—H17C	109.5
C5—C6—H6	120.3	H17A—C17—H17C	109.5
C1—C6—H6	120.3	H17B—C17—H17C	109.5
N1—C7—C1	124.2 (2)	O4—C18—H18A	109.5
N1—C7—H7	117.9	O4—C18—H18B	109.5
C1—C7—H7	117.9	H18A—C18—H18B	109.5
N2—C8—O3	121.5 (2)	O4—C18—H18C	109.5
N2—C8—C9	120.5 (2)	H18A—C18—H18C	109.5
O3—C8—C9	118.0 (2)	H18B—C18—H18C	109.5
